# Collagen-Based Hydrogels for the Eye: A Comprehensive Review

**DOI:** 10.3390/gels9080643

**Published:** 2023-08-09

**Authors:** Dhwani Rana, Nimeet Desai, Sagar Salave, Bharathi Karunakaran, Jyotsnendu Giri, Derajram Benival, Srividya Gorantla, Nagavendra Kommineni

**Affiliations:** 1 National Institute of Pharmaceutical Education and Research (NIPER), Ahmedabad 382355, Gujarat, India; dhwanirana73@gmail.com (D.R.); sagarsalave1994@gmail.com (S.S.); bharathi8599@gmail.com (B.K.); derajram@niperahm.res.in (D.B.); 2 Department of Biomedical Engineering, Indian Institute of Technology Hyderabad, Kandi 502285, Telangana, India; bm21resch11003@iith.ac.in (N.D.); jgiri@bme.iith.ac.in (J.G.); 3 Department of Biomedical Engineering, Tufts University, Medford, MA 02155, USA; gorantlasrividya999@gmail.com; 4 Center for Biomedical Research, Population Council, New York, NY 10065, USA

**Keywords:** collagen, hydrogel, eye, drug delivery, biomedical applications

## Abstract

Collagen-based hydrogels have emerged as a highly promising platform for diverse applications in ophthalmology, spanning from drug delivery systems to biomedical interventions. This review explores the diverse sources of collagen, which give rise to different types of collagen protein. The critical isolation and purification steps are discussed, emphasizing their pivotal role in preparing collagen for biomedical use. To ensure collagen quality and purity, and the suitability of collagen for targeted applications, a comprehensive characterization and quality control are essential, encompassing assessments of its physical, chemical, and biological properties. Also, various cross-linking collagen methods have been examined for providing insight into this crucial process. This comprehensive review delves into every facet of collagen and explores the wide-ranging applications of collagen-based hydrogels, with a particular emphasis on their use in drug delivery systems and their potential in diverse biomedical interventions. By consolidating current knowledge and advancements in the field, this review aims to provide a detailed overview of the utilization of engineered collagen-based hydrogels in ocular therapeutics.

## 1. Introduction

**Figure 1:**
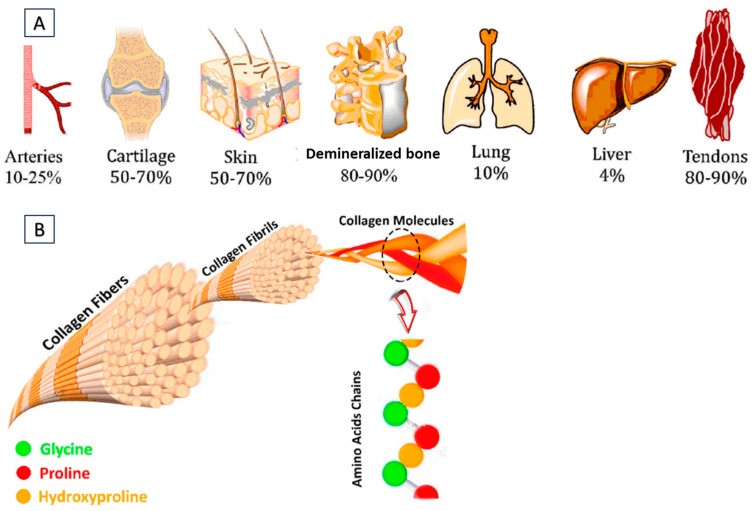
(**A**) Different tissues containing approximate collagen content. (**B**) Representation of collagen fibers, fibrils, triple helices of alpha chains, and amino acid residues. Reproduced with permission from reference [3].

Collagen, a widely recognized extracellular matrix protein, has found extensive use in medical, pharmaceutical, and cosmetic applications. This is due to its crucial role in tissue and organ formation, and its involvement in various cellular functions. Figure 1 demonstrates the approximate collagen content in different tissues. Additionally, collagen serves as an effective surface-active agent and displays its capacity to permeate lipid-free interfaces. In comparison to other natural polymers like albumin and gelatin, collagen showcases exceptional biodegradability, minimal antigenicity, and remarkable biocompatibility. Collagen’s outstanding biocompatibility and safety have led to its rapid and extensive utilization in biomedical applications, particularly in bioengineering [1]. The key advantage of collagen lies in its ability to self-aggregate and cross-link, resulting in the formation of robust and stable fibers. Collagen’s versatility as a drug delivery system is vast and diverse. It can be transformed into different delivery systems upon extracting it into an aqueous solution [2].

Collagen is an extensively utilized biomaterial that serves as a pivotal structural protein within ocular tissues [4]. Collagen-based scaffolds have shown great potential for various biomedical applications, and drug delivery including their use in ophthalmology for eye-related treatments [5,6]. Hydrogels are three-dimensional polymeric networks that are cross-linked, primarily through hydrophilic interactions with water molecules, resulting in a complex structure [7,8]. The unique properties of hydrogels, including hydrophilicity, elasticity, and flexibility, make them highly suitable for various applications such as regenerative medicine, biomaterial engineering, encapsulated cell technology, and drug delivery. One significant advantage of hydrogels is their potential to address the issue of low ocular bioavailability commonly associated with conventional ocular formulations like gels, suspensions, ointments, and eye drops. Hydrogels can enhance the bioavailability of drugs by increasing the drug’s precorneal residence time, allowing for optimal therapeutic action [9,10,11]. This makes hydrogels an important approach in improving drug delivery to the eye. Moreover, hydrogels can exhibit a sol–gel phase transition in response to specific physiological factors (such as ions, enzymes, pH, and temperature) or external stimuli (such as light and electrical current). This responsiveness enables hydrogels to undergo reversible changes in their physical state, facilitating the controlled release of drugs or other therapeutic agents based on the surrounding conditions [12,13]. The desirable properties of functionalized hydrogels have positioned them as potential candidates for ophthalmic applications [14,15]. The exploration of collagen-based hydrogels in ophthalmology holds promise for advancing therapeutic strategies and addressing ocular challenges [16]. Through engineering, collagen-based hydrogels can be tailored to exhibit mechanical properties that are essential for supporting and safeguarding ocular tissues, including appropriate stiffness and elasticity. Recent developments have led to the development of various types of collagen-based hydrogels, either as standalone materials or in combination with other polymers [17]. These combinations with additional polymers can impart specific properties to the final product [18,19]. This review provides an in-depth exploration of collagen, encompassing all aspects of this versatile protein, and offers a comprehensive understanding of the diverse applications of collagen-based hydrogels in drug delivery and various biomedical fields.

## 2. Types of Collagens

Collagen plays a crucial role in the structural integrity of various tissues in living organisms. It is widely distributed throughout the body, spanning from the skin to the cornea of the eye. Collagen serves as a predominant component in several tissues, including skin, bone, tendon, cartilage, blood vessels, and teeth. The diverse functions of collagen in these tissues necessitate the presence of different collagen types, each exhibiting unique interactions with other collagen molecules and the surrounding tissue components. While over 29 types of collagen have been identified, collagen types I to V are the most abundant and extensively studied for their biomedical applications [20]. Type I collagen, also the most abundant type of collagen in the human body, is primarily derived from bovine or porcine sources, specifically from skin, tendons, and bones. Type II collagen, on the other hand, is predominantly located in cartilage, particularly within joint tissues. Type III collagen is commonly found in the skin, blood vessels, and internal organs. As for Type IV collagen, it serves as a key constituent of basement membranes and can be obtained from the human placenta. Finally, Type V collagen, which is mainly utilized in the context of corneal treatment, is found in structures such as hair, cell surfaces, and the placenta [21].

**Figure 2:**
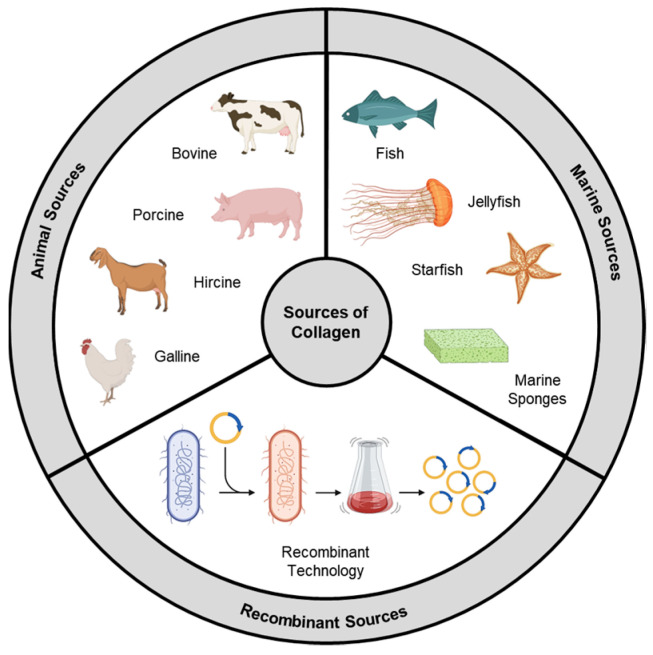
Sources of collagen.

Collagen types exhibit significant variations in their amino acid compositions, leading to distinct chemical and physical properties. These variations impact parameters that govern their biomedical utilization, like thermal stability, solution viscosity, and cross-linking density [22,23]. Within collagen, specific amino acid sequences play a crucial role as substrates for integrins, a class of transmembrane receptors facilitating cell–extracellular matrix (ECM) adhesion. Integrin receptors consist of α and β subunits that form transmembrane heterodimers. The binding specificity demonstrated by various receptors implies that structural differences in collagens derived from different sources will result in functional changes in their interactions [24]. In the past, animal collagen was the primary choice for various biological applications. However, as the field has advanced, other sources like marine-derived and synthetically produced recombinant collagen have gained significant attention (Figure 2). The following section delves into different types of collagens (based on their sources) while also discussing the key attributes that govern their utilization as biomaterials.

### 2.1. Animal Source

Animal collagen is a conventional and predominant source of collagen for biomedical applications. It is derived from various tissues and organs of vertebrate animals, with a primary emphasis on mammals. This source offers several advantages due to its abundance, similarity to human collagen, and suitability for a diverse range of in vivo applications. The livestock industry has been thriving for many decades, and when animals are processed for meat, not all parts are fit for human consumption. Hides and other non-edible parts, including bones, tendons, ligaments, and connective tissues, contain significant amounts of collagen. These collagen-rich byproducts can be processed to extract a significant amount of collagen [25].

Approximately one-third of the protein mass of bovine (beef) species consists of collagen, with tendons containing up to 60–85% collagen [26]. Bovine collagen primarily falls into the category of type I collagen and is characterized by its elongated fibrillar structure, which imparts tensile strength and structural integrity to a variety of tissues. It is composed of two α1 chains and one α2 chain, forming a triple helix conformation. Apart from tendons, type I collagen is abundant in ligaments, bones, and other connective tissues [27]. Type III collagen, on the other hand, is exclusively found in the skin. The yield of collagen during the isolation process varies depending on the age of the bovine tissue, with younger tissues generally yielding higher amounts of collagen. Additionally, the distribution of proteoglycans and post-translational modifications also varies with age, potentially influencing the thermal stability and fibrous self-assembly [28]. Bovine collagen exhibits favorable characteristics of biocompatibility and low immunogenicity. It is generally well-tolerated in vivo and does not induce an immune response in the majority of individuals, except in cases of significant collagen allergies [29]. Porcine (pork) collagen, derived from pigskin, bones, and intestines, can be extracted with a high yield (mainly type I). It exhibits exceptional tensile strength, cell adhesion, and proliferation-promoting properties, rendering it highly suitable as a biomaterial for implantation or reconstructive surgeries [30].

Bovine and porcine collagen are extensively researched and widely used sources of collagen. However, their applicability may be limited in certain regions or among specific populations due to religious beliefs and dietary practices. Other animal-derived collagen sources include ovine (lamb), hircine (goat), equine (horse), and galline (chicken) [31]. While these animal sources can be obtained on an industrial scale and are suitable for commercial applications, among researchers in academia, rat-tail tendon collagen is the most commonly utilized source of type I collagen. The preference for rat-tail tendon collagen in academic research can be attributed to the widespread use of rats as animal models in scientific investigations. The tails of rats are easily accessible and can be isolated after the termination of studies. Rat-tail tendons are particularly advantageous because they contain a high concentration of type I collagen, ranging from 90 to 95% by weight. This characteristic makes rat-tail tendon collagen a convenient, cost-effective, and high-yield source for researchers [32].

### 2.2. Marine Source

The use of mammal-derived collagen is constrained by potential risks associated with immune reactions and the transmission of dangerous zoonotic diseases like bovine spongiform encephalopathies. In recent years, there has been a growing interest in marine organisms as alternative and safe sources of collagen [33]. Biomass derivatives from fish-processing industries and fisheries, including fish and sea urchin waste, undersized fish, and by-catch organisms like jellyfish, starfish, and sponges, hold potential as valuable but currently underutilized sources of collagen [34]. The global per capita consumption of fish has significantly increased (from 9.0 kg in 1961 to 20.5 kg in 2019), resulting in a substantial amount of marine waste, comprising discarded fish parts such as skin, scales, and skeletons (including heads, tails, and fins), as well as low-quality whole fish and non-edible marine species like echinoderms. With approximately 70–85% of the total catch weight being generated as waste or low-value byproducts, there is a compelling incentive to extract valuable bioactive compounds, including collagen, from marine debris to enhance the environmental and social sustainability of the fishing industry [35]. The utilization of fish byproducts as collagen sources not only promotes eco-friendliness but also offers attractive prospects in terms of profitability and cost-effectiveness.

Skin and scales obtained from bighead carp, catla carp (Indian carp), globefish, paddlefish, and Japanese sea bass are preferred sources for marine collagen extraction due to their naturally high collagen content [36,37]. By employing suitable isolation techniques, the yield of collagen extracted from marine sources can reach up to 50% of the dry mass [38]. Marine collagen exhibits comparable biocompatibility and functionality to mammalian collagen, as the genetic sequence of collagen is generally conserved and similar across species. Moreover, marine collagen poses a lower immunological risk to individuals allergic to mammalian products, as marine tissues lack mammalian antigens such as alpha-gal.

Numerous studies have demonstrated that fish collagen, as well as the collagen of most jellyfish species, is characterized by the typical repetitive sequence (Glycine-X-Y)_n_, where X and Y are imino acid residues. Fish collagen exhibits high concentrations of glycine (approximately 30%) and hydroxyproline (8–10%), while levels of histidine, a precursor to histamines associated with allergic responses, are relatively low [39]. The presence of imino acids plays a crucial role in maintaining the structural integrity of collagen. The pyrrolidine rings in imino acids restrict the flexibility of the polypeptide chain, reinforcing the triple helix structure and influencing the thermal stability of the collagen molecule. As a result, the denaturation temperature of collagen increases with the imino acid content [40]. Cold-water fish species typically possess lower imino acid content, resulting in collagen with a lower denaturation/melting temperature (approximately 15–20 °C) compared to warm-water species (approximately 30–35 °C). To enhance the thermal stability of marine collagen, suitable cross-linking treatments can be employed, such as those based on carbodiimide or glutaraldehyde. These treatments help to improve the collagen’s resistance to thermal denaturation [41].

### 2.3. Recombinant Collagen

**Figure 3:**
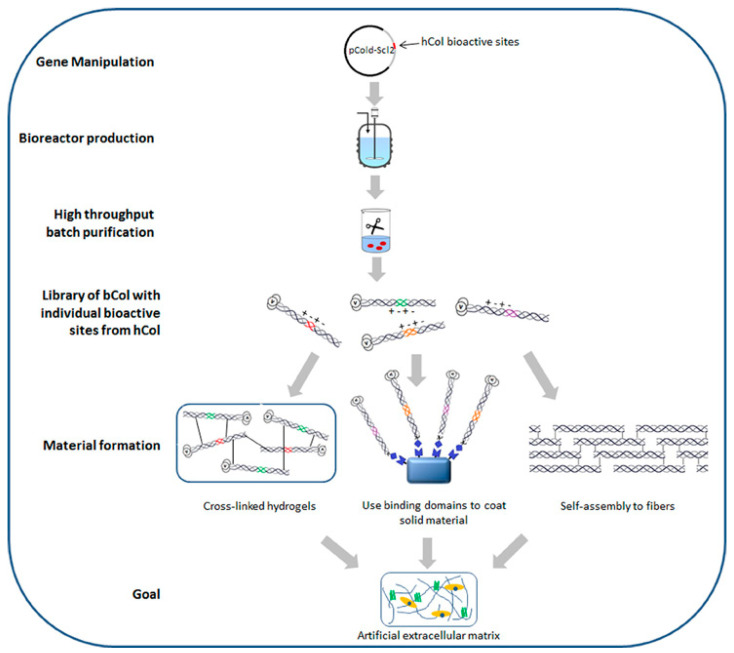
Schematic representation for production of recombinant bacterial collagen and strategies for its material fabrication. Adapted from the reference [44].

Recombinant collagen is produced through genetic engineering techniques using recombinant DNA technology. Unlike animal-derived or marine-derived collagen, recombinant collagen is synthesized in a laboratory setting, offering several advantages and opportunities for customization in biomedical applications. Its production involves the introduction of specific collagen genes or gene fragments into host cells [42]. These host cells are modified to express and produce the desired collagen protein. The introduced genes are often derived from human or animal collagen sequences to ensure the production of collagen that closely resembles native collagen found in living organisms. Uninterrupted large-scale production and precise control over the product’s composition/properties are key advantages [43]. Figure 3 depicts the schematic representation for production of recombinant bacterial collagen and strategies for its material fabrication.

Through genetic engineering techniques, specific modifications can be made to the collagen sequence, allowing for the incorporation of desirable functionalities or targeting specific applications. For example, the introduction of specific amino acid sequences or motifs can promote cell adhesion, enhance tissue regeneration, or improve the stability of the collagen scaffold [45]. Table 1 summarizes different recombinant expression systems reported as sources of collagen for biomedical applications.

The assembly of recombinant collagen into higher-order structures is an essential aspect of its structural properties. Native collagen forms fibrils and networks that contribute to the strength and stability of tissues. Recombinant collagen can also self-assemble into fibrils, mimicking the natural behavior of collagen [47]. The structural properties of recombinant collagen can be further modulated by various post-translational modifications, such as glycosylation or hydroxylation. These modifications, which occur naturally in collagen biosynthesis, can be introduced during the recombinant production process to enhance the stability, functionality, and bioactivity of the collagen protein [48].

The characterization and quality control of recombinant collagen are crucial in its production. Techniques such as SDS-PAGE, mass spectrometry, and circular dichroism spectroscopy are employed to assess the molecular weight, purity, and secondary structure of the recombinant collagen [49]. These analyses ensure that the synthesized collagen meets the desired specifications for its intended application. While recombinant collagen offers numerous advantages, there are also challenges associated with its production. Achieving the correct folding and assembly of the recombinant collagen protein to form the native triple helical structure can be complex. Various strategies, such as the co-expression of molecular chaperones or the use of specialized expression systems, are employed to enhance the proper folding and assembly of recombinant collagen [50].

## 3. Isolation and Purification of Collagen

The isolation and purification of collagen are critical procedures in the preparation of collagen for biomedical applications. These processes entail extracting collagen from its natural sources and eliminating impurities to obtain a highly pure and bioactive form of the protein. The following section provides a comprehensive overview of the individual steps involved in the isolation and purification process, along with key information pertinent to each step.

### 3.1. Pre-Treatment

Pre-treatment is a critical step in the isolation and purification of collagen for biomedical applications. Its purpose is to prepare the source material by eliminating non-collagenous components, surface contaminants, and debris. The selection of the collagen source is determined by the specific requirements of the intended application, emphasizing high quality and freedom from disease or contamination. To initiate the pre-treatment process, the source material undergoes thorough cleaning and washing to eliminate dirt, blood, and external contaminants. Animal tissues, particularly skin and tendons, often contain significant amounts of fat and connective tissue, which can interfere with collagen extraction and purification processes [51]. These components are removed by manually trimming the fat from the tissue and carefully dissecting or stripping away the connective tissue using surgical instruments. Subsequently, the source material is either ground or cut into pieces to increase its surface area, facilitating subsequent extraction steps. Manual methods involving knives or scissors and mechanical techniques using specialized equipment like tissue homogenizers can be employed [52].

It is crucial to maintain tissue integrity during this process, avoiding excessive heat or damage that could compromise the quality of the collagen. If the source material contains a high fat content, defatting becomes necessary to eliminate lipids that can interfere with collagen extraction and purification. Defatting methods include solvent extraction, enzymatic digestion, or mechanical separation. Solvent extraction involves treating the tissue with an organic solvent, such as chloroform, butanol, or ethanol, to dissolve the fat [53]. Enzymatic digestion utilizes lipase enzymes to break down the lipids present. Mechanical separation techniques involve centrifugation or filtration to physically separate the fat from the tissue [54].

In the extraction of collagen from bone, the process of demineralization is essential to eliminate mineral components like calcium and phosphates. This step is crucial, as the presence of minerals can hinder collagen solubilization and impact the quality of the final product. Demineralization techniques involve immersing the bone in acidic solutions, such as hydrochloric acid (HCl) or ethylenediaminetetraacetic acid (EDTA), to dissolve the minerals. The acid solution is periodically changed until the desired level of demineralization is achieved [55]. Disinfection and sterilization measures are implemented following demineralization to ensure the collagen source is devoid of microbial contamination. Disinfection methods may involve treating the source material with antimicrobial agents or solutions like ethanol or iodine-based solutions, which effectively eliminate surface bacteria and fungi. Suitable sterilization techniques, such as gamma irradiation, are employed to eradicate potential pathogens and ensure the safety of the collagen product [56]. The pre-treated material is then appropriately stored to prevent microbial growth and degradation. Typically, it was stored in sealed containers or bags at low temperatures, such as refrigeration or freezing, based on stability requirements.

### 3.2. Extraction

The extraction process involves solubilizing collagen from the source material while preserving its structure and bioactivity. Two of the most widely used extraction methods are discussed below.

#### 3.2.1. Acid Extraction

Acid extraction is widely employed for the extraction of collagen, particularly from skin, tendons, and bones. This process involves the treatment of the source material with an acidic solution to solubilize collagen while denaturing non-collagenous proteins [57]. To initiate the extraction, the pre-treated and finely minced/ground source material is immersed in a chilled acidic solution, typically hydrochloric acid (HCl) or acetic acid, with a concentration ranging from 0.1 M to 0.5 M. The controlled temperature and soaking duration, typically lasting several hours to overnight, allow the acid to break down non-collagenous components and facilitate collagen solubilization [58]. Subsequently, the acidic solution is neutralized to prevent further collagen degradation and adjust the pH to a physiological range. Neutralization agents such as sodium hydroxide (NaOH) or sodium bicarbonate (NaHCO_3_) are commonly employed, with careful pH adjustment monitored using a pH meter. Finally, the resulting collagen solution is filtered to eliminate insoluble debris, including undigested tissue remnants and non-collagenous proteins, ensuring a purified collagen solution.

#### 3.2.2. Enzymatic Digestion

The extraction of collagen from collagen-rich tissues, such as cartilage, is commonly achieved using proteolytic enzymes. This method selectively degrades non-collagenous proteins, facilitating the isolation of collagen. Proteolytic enzymes like trypsin, pepsin, or collagenase are employed, with the choice of enzyme determined by the source material and the desired collagen type. For instance, pepsin is often utilized for extracting acid-soluble collagen, while collagenase is preferred for obtaining type II collagen from cartilage [59]. The optimization of enzyme concentration and incubation time is carried out based on the specific characteristics of the enzyme and tissue [60]. The tissue, minced or finely ground, is then incubated in an enzyme solution under suitable temperature and pH conditions. As the enzyme acts, non-collagenous proteins are broken down, facilitating the release of collagen into the solution. The duration of digestion varies depending on the tissue type and the activity of the enzyme used.

Post extraction, the mixture is filtered to separate the collagen-rich solution from undigested tissue fragments or residual enzymes. Filtration methods such as centrifugation, membrane filtration, or sedimentation are employed to isolate the desired collagen fraction. Mechanical disruption using sonication or high-pressure homogenization can be combined with other above-mentioned methods to enhance collagen yield [61]. Collagen extraction methods can be tailored based on the characteristics and requirements of the source material. Chelating agents, like ethylenediaminetetraacetic acid (EDTA), are effective for extracting collagen from mineral-rich tissues such as bone. These agents facilitate the removal of minerals like calcium and phosphates by chelating metal ions [62].

Alkaline treatment, typically using sodium hydroxide (NaOH), is suitable for extracting specific collagens, like type IV collagen, from basement membranes. This treatment dissolves non-collagenous proteins and liberates collagen [63]. Optimizing the extraction yield and quality often involves employing a combination of different methods. For instance, a sequential extraction process may begin with acid treatment followed by enzymatic digestion to enhance collagen solubilization. Precise optimization is crucial to achieving the desired collagen yield, purity, and quality. Factors such as the concentration and type of extraction reagents, temperature, pH, incubation time, and mechanical forces must be carefully adjusted based on the source material and target collagen type [64]. Continuous monitoring and adjustment of these parameters during processing help preserve the stability, integrity, and bioactivity of the extracted collagen.

### 3.3. Purification

Purification is a vital step in the isolation and preparation of collagen. It involves removing impurities, non-collagenous proteins, and contaminants from the extracted collagen to obtain a pure and bioactive form of the protein. Some commonly used purification methods are discussed below.

#### 3.3.1. Dialysis

Dialysis is a widely used technique in collagen processing, involving the placement of the collagen solution within a dialysis membrane or tubing. The dialysis membrane serves as a semi-permeable barrier, allowing for the selective diffusion of molecules. In this process, the collagen solution is immersed in a dialysis buffer or saline solution. Due to the size difference, small molecules, salts, and impurities can diffuse out of the dialysis bag, while the larger collagen molecules are retained within. This allows for the removal of low-molecular-weight impurities and dialyzable salts from the collagen solution, resulting in a purified and concentrated collagen product [65].

#### 3.3.2. Precipitation

The process of precipitation is based on the principle of inducing collagen to precipitate while leaving impurities behind. This can be achieved by adding alcohol, such as ethanol or isopropanol, to the collagen solution, causing the collagen to precipitate while the impurities remain in the solution. To enhance collagen precipitation, the mixture is typically cooled or refrigerated [66]. After precipitation, the mixture undergoes centrifugation to collect the collagen pellet, which is then washed to remove residual alcohol and impurities. Alternatively, certain salts, like sodium chloride, can be employed to induce collagen precipitation. The collagen solution is mixed with a concentrated salt solution, followed by centrifugation to collect the precipitated collagen. Washing the pellet with a salt-free buffer aids in the removal of residual salts and impurities. Adjusting the temperature or pH can also selectively induce collagen precipitation. Lowering the temperature or adjusting the pH to the isoelectric point of collagen (around pH 4.7–5.0) can cause collagen to precipitate. The resulting collagen precipitate is separated through centrifugation and subsequently subjected to washing steps for further purification, thereby eliminating impurities [67].

#### 3.3.3. Chromatography

Collagen purification often relies on chromatographic techniques, renowned for their exceptional selectivity and capability to separate molecules based on distinctive properties. Several chromatographic methods are applicable to collagen purification, including gel filtration chromatography, ion exchange chromatography, and affinity chromatography [68]. Gel filtration chromatography segregates molecules by size through a column packed with porous beads. As the collagen solution traverses the column, larger collagen molecules elute early, whereas smaller impurities elute later, achieving effective separation. Ion exchange chromatography, on the other hand, separates molecules based on their charge. The collagen solution is loaded onto an ion exchange column functionalized with charged groups. Collagen molecules adhere to the column according to their charge, and the selective elution of collagen while retaining impurities is achieved by employing varying pH or ionic strengths during elution. Affinity chromatography capitalizes on specific interactions between ligands and collagen molecules for purification. Ligands with a high affinity for collagen, such as gelatin or antibodies, are immobilized on a chromatography column. Passing the collagen solution through the column results in collagen molecules specifically binding to the ligands. Through subsequent washing steps to remove impurities, the bound collagen can be eluted using specific elution conditions, ensuring a purified collagen sample [69].

After the purification process, it is often necessary to concentrate the collagen solution to achieve the desired concentration and eliminate excess buffers or diluents. Various methods can be utilized for concentration, such as ultrafiltration and centrifugal concentration [70]. These techniques involve the passage of the collagen solution through a membrane or high-speed centrifugation to concentrate the collagen while retaining impurities and smaller molecules. Simultaneously or separately, desalting can be performed to remove salts or buffer components from the concentrated collagen solution [71]. Desalting can be accomplished through dialysis, gel filtration, or the use of desalting columns that selectively retain salts while allowing the collagen to pass through. Additionally, a final sterilization step may be required to ensure the safety and sterility of the purified collagen. By employing these concentration, desalting, and sterilization processes, the collagen solution can be prepared for various biomedical applications while maintaining its desired properties and meeting the necessary safety standards.

## 4. Characterization and Quality Control

Characterization and quality control involve assessing the physical, chemical, and biological properties of collagen to ensure its quality, purity, and suitability for specific applications.

### 4.1. Physical Characterization

Visual appearance, encompassing color, transparency, and homogeneity, is examined to ensure its desirable attributes. Moreover, the solubility of collagen in various solvents, including water, acid, or alkali, is assessed to gauge its purity and extraction efficiency [72]. The pH value of collagen solutions is measured to verify their compatibility with physiological conditions, while viscosity measurements contribute valuable information regarding collagen concentration and molecular weight. Additionally, particle size analysis techniques such as dynamic light scattering or laser diffraction can determine the distribution of collagen particles, offering insights into aggregation tendencies and aiding in the optimization of formulation and application strategies [73].

### 4.2. Chemical Characterization

The amino acid composition of collagen has been assessed using methods like high-performance liquid chromatography (HPLC) or amino acid analysis. This analysis confirms the presence of glycine, proline, and hydroxyproline, which are highly abundant in collagen. Secondly, the purity and molecular weight distribution of collagen can be evaluated using Sodium Dodecyl Sulfate-Polyacrylamide Gel Electrophoresis (SDS-PAGE). This method provides information about impurities and confirms the presence of specific collagen types [74]. Fourier Transform Infrared Spectroscopy (FTIR) is another valuable technique used to study the chemical structure and conformation of collagen. It detects characteristic absorption peaks, such as amide I and amide II bands, which indicate the presence of collagen and its secondary structure [75]. Additionally, circular dichroism (CD) spectroscopy has been employed to analyze conformational changes and secondary structure. CD spectroscopy allows for the assessment of the triple helical structure, stability, and folding of collagen molecules [76].

### 4.3. Biological Characterization

Biocompatibility assessments involve both in vitro and in vivo assays to determine how well collagen interacts with living tissues and cells. In vitro assays measure cell viability, proliferation, adhesion, and migration to evaluate cytocompatibility and biological response. Immunogenicity studies examine the immune response triggered by collagen, employing assays like ELISA and immunohistochemistry to detect immune reactions, antibody production, and inflammatory responses [77]. Bioactivity assessment focuses on collagen’s capacity to facilitate cell attachment, migration, proliferation, and tissue regeneration. In vitro assays with relevant cell types, such as fibroblasts or endothelial cells, are conducted to explore the bioactive properties of collagen [78].

### 4.4. Microbiological Quality Control

Sterility testing is performed to verify the absence of microbial contamination. This involves employing standard microbiological techniques, such as culture-based methods or membrane filtration, to evaluate the microbial load present in the collagen samples. Additionally, endotoxin levels are determined through the use of the Limulus Amebocyte Lysate (LAL) assay, which specifically detects the presence of bacterial endotoxins. This testing is essential to ensure that the collagen meets regulatory requirements for endotoxin levels, thus ensuring the safety of the product for its intended use [79,80].

### 4.5. Stability Studies

Stability studies are crucial for determining the shelf life and stability of collagen products. Shelf-life determination involves subjecting collagen samples to specific storage conditions while monitoring their physicochemical and biological properties over time [81]. Parameters such as collagen degradation, changes in molecular weight, and loss of bioactivity are assessed to establish the product’s shelf life. Accelerated aging tests simulate long-term storage effects in a shorter time frame by exposing collagen samples to elevated temperatures, humidity, or other stress conditions [82]. This helps assess the stability and degradation kinetics of the collagen product. Freeze–thaw stability tests evaluate the product’s stability during storage and transportation by subjecting collagen formulations to multiple freeze–thaw cycles. The physical and chemical properties of the collagen samples are assessed to ensure their integrity and stability. Collectively, these stability studies provide valuable insights into the long-term viability and suitability of collagen products for various applications.

### 4.6. Quality Control and Regulatory Compliance

To ensure reproducibility and reliability, collagen products must demonstrate batch-to-batch consistency. Quality control measures are implemented to monitor key parameters, including physical appearance, chemical composition, and biological activity, across different batches [83]. Standardization is essential to define and control critical quality attributes such as purity, collagen type, and bioactivity. Specifications and acceptance criteria are established to maintain consistency and meet regulatory requirements. Traceability is also important, as proper documentation and tracking of collagen batches help identify the source of any deviations or issues during production or post-production. Regulatory compliance is vital, and collagen products must adhere to guidelines such as Good Manufacturing Practices (GMP), ISO standards, and relevant regulatory frameworks [84]. By implementing stringent quality control measures and adhering to regulatory guidelines, the collagen industry can ensure the production of high-quality and reliable collagen products for biomedical use.

## 5. Types of Cross-Linking Methods in Hydrogel Synthesis

The two key strategies by which hydrogels can be fabricated include physical and chemical cross-linking. The physical cross-linking strategies make use of crystallization, hydrogen bonding, ionic/electrostatic interactions, and hydrophobic interactions formed either via thermal induction based on the lower critical solution temperature (LCST)/upper critical solution temperature or through ultrasonication-based sol–gel transitions. In contrast, the chemical cross-linking approaches include enzyme-induced cross-linking, photo-polymerization, and click-chemistry reactions such as the Michael-type addition, oxime formation, Schiff base formation, and Diels–Alder “click” reaction [85]. The below section summarizes the role of these strategies in the design and fabrication of novel hydrogels with desirable characteristics.

### 5.1. Physically-Cross-Linked Hydrogel

The intermolecular reversible interactions responsible for the formation of physically cross-linked hydrogels possess a major advantage over chemical cross-linking methods, due to the lack of utilization of any chemical agents that may lead to potential cytotoxicity. The selection of the type of polymer is an important factor in hydrogel synthesis, as it determines the strength of interactions among the various chains that constitute the molecular network, which in turn determines the water-holding capacity of the hydrogels [86].

#### 5.1.1. Cross-Linking by Hydrophobic Interactions

The polymers that possess both hydrophilic and hydrophobic groups exhibit thermally induced phase transitions, which are responsible for the cross-linking of hydrogels. At lower concentrations, the amphiphilic copolymers self-assemble in aqueous solutions to form micelles with a hydrophobic core. On increasing the temperature, the nearby micelles tend to undergo re-aggregation through hydrophobic interactions, resulting in gel formation. These thermo-responsive polymers remain in the soluble form below the lower critical solution temperature, and become insoluble through hydrophobic interactions above the LCST. Examples of such thermo-responsive polymers include Poly(N-isopropylacrylamide), and several copolymers containing a combination of the hydrophilic polymer poly(ethylene oxide) with hydrophobic polymers such as poly(glycolide), poly(propylene oxide), poly(lactide), and poly(ε-caprolactone) [87]. Such polymers are being extensively explored as drug delivery systems due to their ability to be present as injectable solutions at room temperature, that then undergo a switch to the gel form at body temperature. Blending collagen with such synthetic polymers tends to increase its mechanical strength, which has been widely explored in tissue engineering applications. On the contrary, several polymers remain in the solution form at higher temperatures, and upon cooling below the upper critical solution temperature (UCST) induce the formation of hydrogel. These hydrogels formed by micellar aggregation below the UCST tend to disintegrate upon restoration of the UCST, as the polymers become water-soluble in nature [88].

#### 5.1.2. Cross-Linking by Ionic/Electrostatic Interactions

The electrostatic interactions that occur between two macromolecules of opposite electric charges are extensively utilized for the physical cross-linking of hydrogels. Such hydrogels can be subjected to a wide range of modifications, including changes in the charge density of the polymer, concentration of the polymer, ratios of mixing, and the soluble microenvironment of the polymer, in order to obtain the desired characteristics. An example of physical cross-linking through electrostatic interactions is the cross-linking of the negatively charged polysaccharide alginate by divalent cations, such as magnesium (Mg^2+^), calcium (Ca^2+^), and barium (Ba^2+^). In the case of collagen hydrogels, cross-linking confers enhanced mechanical strength to the macromolecular network. The process of self-assembly of collagen monomers into fibrillar structures, and the subsequent cross-linking to form viscoelastic gels, is known as fibrillogenesis. The various factors that affect fibrillogenesis include pH and salt concentrations. It has been reported that changes in the ionic strength lead to alterations in the fibril diameter, thereby suggesting the role of electrostatic interactions in the lateral growth of collagen fibrils [89].

#### 5.1.3. Cross-Linking by UV Irradiation and De-Hydrothermal (DHT) Treatment

Cross-linking of collagen can be achieved through external physical factors such as de-hydrothermal treatment or UV irradiation, which successfully modulate the microstructural organization and mechanical properties of collagen. DHT treatment involves subjecting the collagen scaffolds to a high temperature of around 90 °C in a vacuum oven, which causes the intermolecular cross-linking of collagen through condensation of the carboxylic acid and amino acid side chains, whereas irradiation with UV cross-linking occurs through the production of free radicals on the phenylalanine and tyrosine residues. However, a reduction in the stiffness of the collagen matrix has been documented with increasing UV irradiation time, suggesting the degradation of the matrix. DHT treatment is a comparatively faster method of cross-linking than UV irradiation, which reduces the time of treatment from days to minutes [90].

### 5.2. Chemically-Cross-Linked Hydrogel

The formation of covalent bonds between polymeric chains is the key mechanism behind the synthesis of chemically cross-linked hydrogels. These networks, in comparison to physically cross-linked hydrogels, tend to exhibit enhanced stability upon exposure to physiological conditions, as well as tuneable degradation behavior. Further, certain characteristics, such as enhanced mechanical strength, make it a widely used technique for the cross-linking of hydrogels. A wide range of cross-linkers, such as carbodiimides, glutaraldehyde, polyepoxy compounds, hexamethylene diisocyanate, and genipin have been explored for the preparation of cross-linked collagen hydrogels, in tissue engineering and drug-delivery applications. The cross-linking efficiency, based on the pH, temperature and concentration of the reagent, determines the number of amino acid residues that can react with the cross-linker, the stability of the cross-linker, and the bond energy that is associated with each cross-link [91].

#### 5.2.1. Carbodiimides

1-ethyl-3-(3-dimethyl aminopropyl) carbodiimide hydrochloride and cyanamide are examples of the widely used carbodiimides that belong to the class of zero-length cross-linkers. These cross-linkers exert their action through the formation of peptide-like bonds between the amino and carboxyl groups in collagen. Cross-linking with carbodiimides occurs with maximum efficiency at a mildly acidic pH of about 4.5. However, it can also take place under physiological conditions (pH 7.4, 37 °C), making it an attractive option for biomedical applications. Further, the chemical reaction is safe, as it yields a non-toxic byproduct, urea, that can be easily washed away after cross-linking [92].

#### 5.2.2. Polyethylene Glycol

The biocompatibility, versatility, low-protein adsorption capacity, and non-immunogenicity of polyethylene glycol have established a long history of its safety in vivo. The linear or branched structures of polyethylene glycol constituting end hydroxyl groups can be functionalized with a wide array of groups, including azides, maleimide, and acrylates to achieve different kinds of cross-linking. The tuneable characteristics of PEG allow optimization of the collage gels, without any deleterious effect on cell viability. Further, star polymeric structures of PEG composed of three-dimensional hyperbranched structures, in which different molecular weights of the polymer are located on the linear arms arising from a central core, may be utilized in various biomedical applications, owing to their ability to hold a high number of functional groups in a small space [93].

In a study by Sargeant et al., injectable hydrogels composed of collagen characterized by multi-armed polyethylene glycol were fabricated for tissue regeneration applications. While the collagen component promoted cellular adhesion, the presence of amine-reactive chemistry in the multi-armed polyethylene glycol structure was found to promote tissue binding. The mechanical strength, swelling, and degradation profile of the hydrogel scaffolds demonstrates the promising potential of collagen hydrogels in the treatment of a variety of tissue defects [94].

#### 5.2.3. Genipin

Natural cross-linking agents, such as genipin, obtained from the gardenia fruit, have gained wide attention in tissue engineering applications owing to their non-cytotoxicity. Genipin undergoes a non-specific reaction with primary amine groups to produce a secondary activated form of genipin, whose ester groups in turn react with proteins through the formation of a secondary amide bond. In a study by Macaya et al., genipin collagen gels were found to exhibit high resistance to degradation by the enzyme collagenase. Also, the physical characterization studies revealed that the addition of genipin to collagen provides control over the mechanical and degradation behavior of the hydrogel, making it an ideal injectable biopolymer for drug delivery applications. The study evaluated the effect of the encapsulation of mesenchymal stem cells in genipin–collagen gels, and it was found that the gels supported cell viability and proliferation, thereby establishing their potential as a promising therapeutic agent in spinal cord injury [95].

## 6. Applications of Collagen-Based Hydrogels

The key goals of designing an ophthalmic drug delivery system are to enhance the bioavailability of drugs, i.e., to deliver therapeutic doses with minimal wastage, avoiding systemic adsorption, and encouraging patient compliance [96]. During the past few years, most of the research studies have reported the substantial potential of hydrogels in delivering therapeutics for effective chronic ocular disease treatment. Among the hydrogel-forming biomaterials, collagen has diverse applications in delivery systems because of its biocompatibility, low immunogenicity, and its easily modified nature into various forms. Also, it is an excellent substrate for the in vitro growth of corneal epithelial cells. Thus, it is an effective drug, protein, and gene delivery carrier [97].

### 6.1. Collagen-Based Hydrogels as Corneal Shields and Collasomes

Numerous researchers have explored the use of external collagen to safeguard the eye’s surface in various disease conditions, leveraging the fact that collagen is a naturally occurring protein widely present in the body and crucial for supporting and protecting vital structures. This approach aims to harness the inherent properties of collagen to promote the healing of wounds and provide protection to critical components, such as the white sclera, clear cornea, and other ocular tissues [98]. The primary application of collagen in ophthalmology is collagen shields [2]. These collagen shields were first developed by Dr. Svyatoslav Fyodorov as postoperative corneal bandages and as a delivery device [99]. BIO-Cor^®^ is a collagen corneal shield device introduced by Bausch + Lomb Pharmaceuticals, Tampa, FL. The developed BIO-Cor^®^ is made up of porcine scleral collagen and cross-links completely within 72 h after insertion into the ocular region [100]. The BIO-Cor^®^ basic mechanism involves hydration of the administered shield by tears, which releases the hydrophilic drug in the cornea and aqueous humor for an extended period of time. In contrast, hydrophobic drugs are directly loaded into the collagen corneal shield during the preparation. Drugs such as prednisolone, cyclosporine, fluorescein, and ofloxacin have been successfully delivered to the anterior segment of the eye [101]. Furthermore, the application of collagen shields has gained significance by delivering plasmid DNA into the bleb after glaucoma surgery for regulating wound healing [102].

The designed collagen corneal shields need an expert physician to insert, and often produce patient discomfort by interfering with vision. Then, the collasomes and lacrisomes were developed, which are collagen-based drug delivery systems used to load the hydrophilic drug, i.e., small collagen pieces suspended in artificial tear preparations. The major benefit of collasomes was due to the suspension of collagen particles in a vehicle, which can be administered as drops and ointment beneath the eyelids without interfering with the vision [103].

Bausch + Lomb Pharmaceuticals (Tampa, FL) manufactured two forms of collasomes using processed collagen (particles of 1 mm × 0.1 mm × 2 mm in size). Among these, one was uncross-linked collagen and the second was a 1:1 mixture of cross-linked and uncross-linked collagen, which showed a 12 h and 24 h dissolution rate, respectively. Additionally, in keratoconjunctivitis sicca patients, the corneal epithelial surface is typically aberrant, and the collagen shields used dissolve slowly, providing sustained levels of lubricant to the cornea. It has also been reported that lacrisome’s lubrication properties are augmented by a combination of long-chain hydrocarbon alcohol with collagen [103,104].

### 6.2. Collagen-Based Hydrogel for Corneal Regeneration

**Figure 4:**
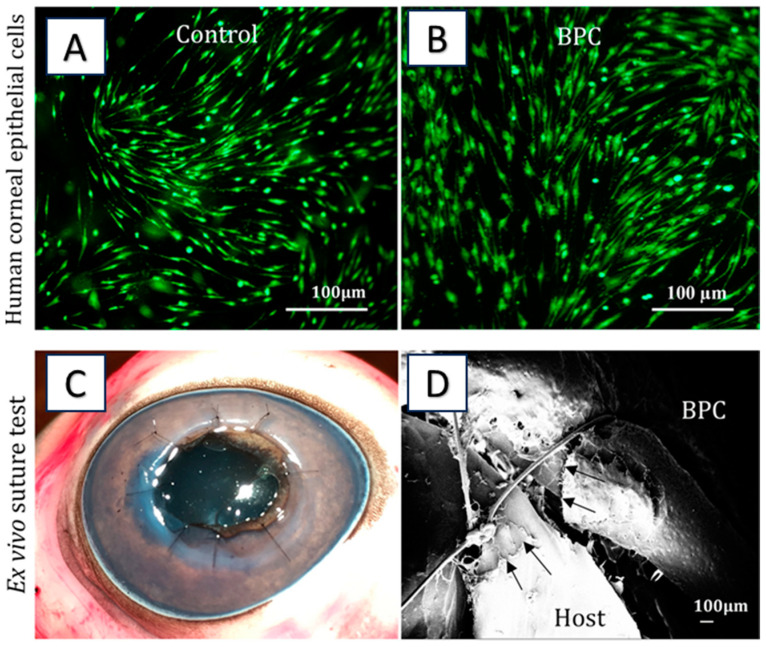
(**A**,**B**) Cytocompatibility studies. (**C**) Ex vivo suturing of BPC into porcine eye. (**D**) SEM images for the intervention of sutured hydrogel with host cornea. Reproduced from reference [17].

Collagen is a major component of the corneal stroma, providing structural support and maintaining corneal transparency [105]. The utilization of a hydrogel based on collagen enables the replacement of a significant portion of the corneal stroma that has been damaged or affected by disease. Collagen-based hydrogel facilitates the regeneration of the cornea by creating a framework for the migration and colonization of host cells and tissues. By enabling the integration of biomaterials with the surrounding tissue, it enables and enhances the process of regeneration. Xeroudaki et al. fabricated a collagen-based hydrogel (BPC) with suitable mechanical properties and transparency to replace damaged or diseased corneal stroma. Moreover, a hybrid surgical implantation technique was also developed, inspired by LASIK refractive surgery, to promote rapid wound healing and maintain the integrity of the hydrogel. The hydrogel exhibited excellent biocompatibility, facilitating cell adhesion, proliferation, and differentiation (Figure 4). Notably, in vivo experiments demonstrated seamless integration of the hydrogel with host tissue and cells, offering optimistic prospects for corneal regeneration. The findings suggest that collagen-based hydrogel holds significant potential for addressing corneal blindness and enhancing visual outcomes. The successful integration of the hydrogel with host tissue, its transparency, and the potential for sustained drug delivery make it a promising candidate for further development and potential clinical application [17].

**Figure 5:**
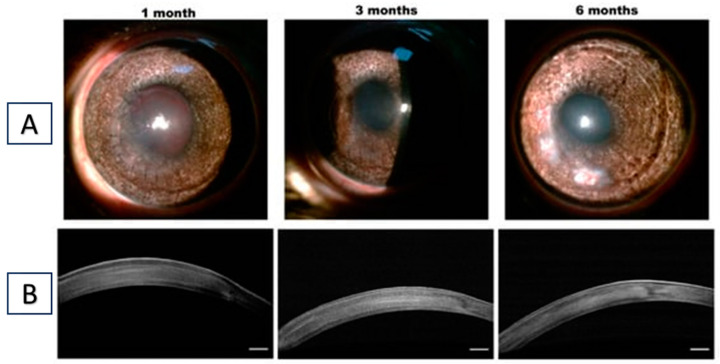
(**A**) Representative photographs at different follow-up times after the implantation of collagen membrane in the stroma. (**B**) OCT images of rabbit corneas. Scale bar = 250 μm. Reproduced from reference [106].

Likewise, Osidak et al. investigated the potential of a collagen-based hydrogel as an artificial stroma equivalent for corneal stroma regeneration. The collagen-based hydrogel implant was well tolerated by the rabbits, with no observable toxic effects or inflammation. The implant was fully integrated into the corneal stroma, and host cell migration within the material was observed. The cornea was preserved throughout and was of uniform thickness, with no visible changes throughout its thickness. The study suggests that collagen-based hydrogel has potential as an artificial stroma equivalent for corneal stroma regeneration, with promising results in terms of biocompatibility and integration into the corneal stroma [106] (Figure 5). The studies underscore the promising prospects of collagen-based hydrogels for corneal regeneration applications.

### 6.3. Collagen-Based Super Macroporous Cryogels

Cryogels are the macroporous heterophase polymeric gels formed through the cryo-gelation of apposite monomers or polymeric precursors at subzero temperatures. For the fabrication of ocular drug delivery devices, collagen-based biomaterials have been widely exploited because of their cell-binding motif, biocompatibility, and tissue-adhesive nature. Collagen-based punctual plugs, i.e., VeraC7TM collagen punctal plugs, have been used for the treatment of dry eye symptoms. The punctal plugs are tiny medical devices inserted in the puncta region of the lacrimal ducts, which can release drugs in a controlled manner, reduce dose, and encourage patient compliance [107].

However, the currently available punctal plug uses the soaking method, which leads to low drug loading. Thus, in a recent study, a punctal plug was fabricated using a cross-linked super macroporous collagen type 1 cryogel with 4s-arm-PEG succinimidyl glutarate by a cryo-gelation technique. Further, moxifloxacin-loaded hyaluronic-acid-based microneedles were loaded into the collagen type 1 cryogel. Interestingly, the results demonstrated rapid drug release initially due to the hydrophilicity of the hyaluronic acid polymer and swelling of collagen, and a slower diffusion of the drug was attributed to hyaluronic acid microneedles [108]. There is a need to conduct in vivo animal studies of the developed hyaluronic-acid-incorporated collagen cryogel for ocular drug delivery.

### 6.4. Collagen-Based Nanocarrier-Loaded Hydrogels

Particulate drug delivery systems are the macromolecular materials that act as drug carriers and vaccine adjuvants. In these systems, the therapeutic agent is encapsulated, entrapped, and dissolved, with key principles such as absorption, adsorption, or attachment. The microparticles and nanoparticles possess similar characteristics, but due to the size effect, they exhibit different optical, electrical, thermal, and magnetic characteristics. The particles that have approximate physical dimensions of between 1 and 1000 μm are considered microparticles [109]. These microparticles improve precorneal residence time and can be cleared by healthy eyes within 50 days, and vitrectomised eyes can clear them within 14 days [110]. The nanoparticles possess three orders of smaller magnitude (<1 m). The nanoparticles offer controlled and sustained drug release of drugs and are retained in the cul-de-sac after topical administration. It has been well reported that nanoparticle-entrapped drugs could be released from the particles at an appropriate rate efficiently by cross-cell membranes and epithelial barriers with distinct internalization mechanisms.

In reported research studies, metallic nanoparticles with a small size of 20 nm have been able to cross the blood–retina barrier with ease. Most importantly, zinc oxide (ZnO) nanoparticles have gained attention [111]. On the other hand, collagen hydrogel cross-linked with polyvinylpyrrolidone-capped ZnO loaded with dexamethasone has exhibited a significant increase in mechanical (adhesiveness and hardness) and physical (viscosity) properties of gel compared to a simple hydrogel [112]. Usually, infectious ocular inflammation conditions are major causes of visual impairment. Thus, the in vivo activity of this cross-linked metallic nanoparticle hydrogel on an ocular inflammatory animal model needs to be evaluated.

**Figure 6:**
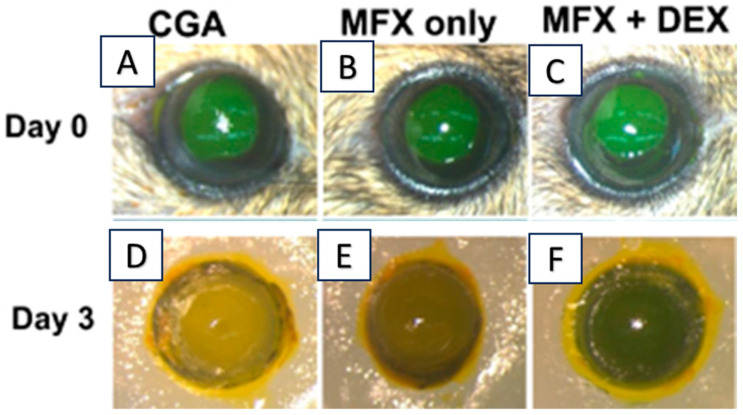
(**A**–**C**) Representative photographs of fluorescein-stained eyes; (**D**–**F**) representative photographs of re-epithelialization after three days’ treatment with different study groups. Adapted with minor changes from reference [124].

In addition, the administration of a biodegradable matrix, including nanoparticles loaded with a potent drug, to the corneal surface has been found to be an appropriate option for treating inflammatory cases and for preventing postoperative infection in patients. Liposomes are termed a versatile drug delivery system [113,114,115,116,117,118,119,120,121,122,123]. Chang et al. developed liposomes loaded with moxifloxacin (MFX) and dexamethasone (DEX) and mixed them with hydrogel-forming biodegradable materials (collagen/gelatin/alginate), i.e., CGA-Lipo-MFX/DEX, for prolonged ocular delivery [124] (Figure 6).

The in vivo efficacy of the above-developed formulation was evaluated on the C57BL/6 corneal epithelial debridement mouse model (Figure 6). The CGA-Lipo-MFX/DEX formulation has exhibited a dual effect, and significantly (*p* < 0.05) reduced the infiltrating leukocytes compared to CGA-Lipo-MFX [124]. These reported studies confirmed that the modification of the mechanical hydrogel properties and loading of liposomes into hydrogels avails researchers the prospect to tailor and sustain drug release, and minimizes adverse responses.

## 7. Concluding Remarks

Biopolymers have emerged as a leading focus in ocular drug delivery research. Collagen-based hydrogels hold great potential in biomedical applications, particularly in the field of drug delivery. By understanding the structural characteristics of collagen and its versatile nature, researchers can leverage collagen-based hydrogels to develop innovative and effective drug delivery systems. While there have been notable advancements in the engineering and modification of hydrogels for ocular drug delivery and tissue engineering, certain important issues must be addressed to ensure the long-term safety, maximize the utility, and improve clinical outcomes of ocular hydrogels. Continued advancements in this field will contribute toward the development of improved therapeutic interventions and biomedical solutions, leading to substantial benefits for patients and propelling the field of biomedical research forward.

## Figures and Tables

**Table 1:** Summary of recombinant expression systems. Adapted and recreated with permission from [46].

Expression System		Transduced Gene	Expressed Collagen
Prokaryote	*Escherichia coli*	COL1A1	Type I
	*Escherichia coli*	COL3A1	Type III
Yeast	*Pichia pastoris*	COL3A1, PH4A/B	Type III
	*Saccharomyces cerevisiae*	COL3A1, PH4A/B	Type III
Human cell lines	HT1080 fibrosarcoma cells	COL1A1	Type I
	HEK 293 kidney epithelial cells	COL5A1	Type V
Mammal	*Mus Musculus* (Mammary gland)	COL1A1	Type I
Insect	*Spodoptera frugiperda* (Sf9 cells)	COL3A1	Type III
	*Bombyx mori*	COL1A1	Gly-X-Y collage-like homodimer

## Data Availability

Not applicable.
